# A Call to Action. A Critical Review of Mental Health Related Anti-stigma Campaigns

**DOI:** 10.3389/fpubh.2020.569539

**Published:** 2021-01-08

**Authors:** Daniel Alexander Benjamin Walsh, Juliet Louise Hallam Foster

**Affiliations:** King's College London, London, United Kingdom

**Keywords:** public health campaigns, implicit, emotion, mental illness, public health education and health promotion, contact theory, stigma, mental health

## Abstract

Using a knowledge-attitudes-behavior practice (KABP) paradigm, professionals have focused on educating the public in biomedical explanations of mental illness. Especially in high-income countries, it is now common for education-based campaigns to also include some form of social contact and to be tailored to key groups. However, and despite over 20 years of high-profile national campaigns (e.g., Time to Change in England; Beyond Blue in Australia), examinations suggest that the public continue to Other those with experiences of mental ill-health. Furthermore, evaluations of anti-stigma programs are found to have weak- to no significant long-term effects, and serious concerns have been raised over their possible unintended consequences. Accordingly, this article critically re-engages with the literature. We evidence that there have been systematic issues in problem conceptualization. Namely, the KABP paradigm does not respond to the multiple forms of knowledge embodied in every life, often outside conscious awareness. Furthermore, we highlight how a singular focus on addressing the public's perceived deficits in professionalized forms of knowledge has sustained public practices which divide between “us” and “them.” In addition, we show that practitioners have not fully appreciated the social processes which Other individuals with experiences of mental illness, nor how these processes motivate the public to maintain distance from those perceived to embody this devalued form of social identity. Lastly, we suggest methodological tools which would allow public health professionals to fully explore these identity-related social processes. Whilst some readers may be frustrated by the lack of clear solutions provided in this paper, given the serious unintended consequences of anti-stigma campaigns, we caution against making simplified statements on how to correct public health campaigns. Instead, this review should be seen as a call to action. We hope that by fully exploring these processes, we can develop new interventions rooted in the ways the public make sense of mental health and illness.

## Introduction

In 2001, the World Health Organization declared that “*the single most important barrier to overcome in the community is the stigma and associated discrimination toward persons suffering from mental and behavioral disorders”* [(1), p. 98]. Since then, public health professionals have predominately followed a deficit model of health-related behaviors (2), and assumed stigma to be maintained by the public's lack of, or incorrect “knowledge” about mental illness (3–5). Accordingly, the majority of interventions have been education-based (3, 6), of which half were stand-alone interventions to promote mental health literacy (MHL) (7–11), and a further third included some form of contact (7, 11–13). In line with a common mental health treatment gap, more than four in five interventions have been conducted in high income countries (11, 13).

Whilst at the population level anti-stigma campaigns have been shown to have small to medium short-term benefits in positive attitude change (7, 13), and it is hoped these attitudinal effects may be sustained (11, 14), there is a serious lack of evidence for long-term behavioral change (13, 15). Furthermore, the unintended effects of these programs have been of particular concern, especially those which exclusively focused on educating the public in biomedical models of mental illness (3, 4). Such models have been found to promote categorical beliefs of difference amongst the public (16–19), and distance-promoting emotions of fear and pity (20–22).

These unintended effects fit into a wider literature on health and stigma, which suggests that the public response to health conditions often follows a common affective distancing-blame-stigma pattern (23). Specifically, examinations of the public's motivations for maintaining health-related stigmas find beliefs of difference to be psychologically pacifying, as they allow those without a form of health condition to perceive themselves both to be invulnerable to the perceived threat and to maintain positive forms of social identity (21, 23–26). However, to our knowledge, no mental health-related public health campaigns have explicitly been designed to challenge these distancing-blame-stigma patterns.

To understand these limited and unintended effects, this review diverges from the dominant approach followed by other reviewers. Namely, since Corrigan et al. (27) published their seminal meta-analysis there have been a number of high-profile reviews, each evaluating the relative effectiveness of education- and contact-based interventions [e.g., (13, 28)]. In these reviews the relative effectiveness of interventions was almost exclusively evaluated using a KABP paradigm (4). However, limited consideration was paid to whether this paradigm effectively responds to the ways the public make sense of mental health and illness.

This review enriches the literature by following an alternative approach. Namely, we show that researchers may have fallen into the trap known as “type III errors” (29, 30). This is when there are systematic issues in a problem conceptualization (29), as is common in health policy (2, 30). In the health promotion domain common indicators of type III errors include: an undue focus on individual-level cognitions; an under-consideration of structural influences; the neglect of lay and service-user forms of expertise; and interventions with significant but mixed and unintended effects (30–33). By reviewing the ways public health professionals have conceptualized and operationalized mental health related stigma, as well as explaining the mixed-effectiveness of these campaigns, we evidence the need to develop new interventions rooted in the ways the public make sense of mental health and illness.

## Mental Health Related Stigma

Public health campaigns have largely conceptualized mental health related stigma as a lack of symmetry between public and professional understanding (3–5). However, within the “*psy disciplines”* [(34), p. 2], there is by no means consensus about what professional understanding should be, as disagreements about what are “typical,” “positive,” or “ordered” forms of behavior are as old as the disciplines themselves (35–38). Nor does holding a form of professional understanding inherently indicate a lack mental health-related stigma, as the history of these of disciplines are intimately interwoven with practices of coercion, violence, and separation (36, 39, 40). Indeed, interventions increasingly focus on challenging professional forms of mental health-related stigma (41–43).

In the absence of a consensual definition, in this section we will describe the common ways professionals have conceptualized mental health-related stigma. Researchers have argued that stigma is a multi-dimensional concept including the co-occurrence of group-based differences, status-loss, social distancing, negative affect, prejudice, and discrimination (28, 44, 45), and that these co-occurrences emerge at multiple levels linking individual expressions of stigma to wider structural and cultural processes (28, 45, 46).

From these broad and multi-dimensional models, public health professionals have typically reduced mental health related stigma into a linear KABP paradigm (4, 28), in which the individual is considered the primary unit of analysis (6, 8, 11, 13, 46). Specifically, they have considered mental health-related stigma to be an individual's lack of professional knowledge, their negative outgroup attitudes, and their desire for social distance from someone perceived as having a mental health problem (3–5).

In part this particularisation reflects some of the agendas involved in their formulation. Specifically, as Corrigan (3) explains that there are three competing agendas involved in the definition of mental health related stigma: (2) a services agenda, which focuses on reducing label avoidance to encourage engagement in evidence-based services; (3) a rights agenda, which focuses on minimizing negative representations of mental illness; and (4) a self-worth agenda, which focuses on encouraging pride for those with experiences of distress.

Reflecting the central role mental health professionals and the pharmaceutical industry have had in the design and financing of anti-stigma campaigns (39, 47), public health campaigns have predominately prioritized a services agenda. The services agenda often draws upon the classic labeling approaches for defining stigma. Namely, it considers stigma to be “*an aversive or hostile attitude toward a person who belongs to a group, simply because he belongs to that group, and is therefore presumed to have the objectionable qualities ascribed to the group* [(48), *p. 7].”* However, the services agenda diverges from these traditional formulations of stigma in an important way: they often reinterpret “objectionable” to be synonymous with ‘inaccurate’. Accordingly, to tackle negative public attitudes, they often focus on creating symmetry between professional and public forms of knowledge (4). They pursue this on the assumption that if there is symmetry in forms of knowledge, potential service users would not avoid stigmatizing labels, and would engage effectively with professional services (3).

Expanding upon this, it is important to note that those promoting a services agenda have a particular understanding of mental illness and stigma. Namely, potential service-users are held in opposition to the “normal” majority; they are considered to hold deficient knowledge about mental illness; and their symptoms are largely considered to reflect an underlying form of biological disorder (4, 40, 49, 50). Indeed, this agenda typically prioritizes biogenetic and neurological explanations of mental illness (3, 40).

In contrast to the services agenda's singular focus on access to professional services, advocates of a rights agenda emphasize the asymmetries in social, economic, and political power that imbue components of stigma with discriminatory consequences (51). In many ways, those pursuing a rights agenda prioritize a classic understanding of mental health-related stigma as a form of stigmata: the marks which reduce those with an undesired label to a lower social status (40, 44, 45, 52). Accordingly, in contrast to those who advocate a services agenda, advocates of a rights agenda place a greater emphasis on explaining service user experiences of distress in terms of societal prejudices rather than barriers to professional services (3, 5).

The self-worth agenda has traditionally had limited influence on the design of national public health campaigns (3). It is primarily concerned with challenging the internalization of stigma (3). To do this, those with experiences of distress develop and operate mutual help and peer support programs (3). These programmes which traditionally tend to favor grassroots participation (53). Although the self-worth agenda and rights agenda both highlight the societal aspects which maintain discrediting experiences of stigma, the self-worth agenda places a greater emphasis on locating stigma within everyday experiences (3). Furthermore, in contrast to the services agenda, a self-worth agenda often takes a broader and potentially more critical approach to psychiatric orthodoxy (3, 39, 47). That is, experiences of distress are considered to be meaningful responses that can only be understood with reference to an individual's life history and their particular social, cultural, and familial contexts (49, 50).

To note, this review will be principally concerned with what in the literature is often referred to as public-, community-, or cultural-stigma (54), labels used to “*mark the nature of the contextual climate of prejudice and discrimination*” [(45), p. 94]. In recent years there has been a focus on distinguishing forms of stigma, such as those which compare between public-, self-, and provider-based stigma (12, 45). Although it is very much in evidence that there may be important differences in understandings between those with and without experiences of the mental health services (55, 56), it is important not to consider public forms of stigma as fully divorced from other forms of stigma (45, 51); a consideration that is often advanced by the self-worth agenda (3). Indeed, as will be discussed later on, in part it is assumptions of categorical differences between those with- and without- a label of mental illness (36) that sustains aspects of public stigma.

## Public Health Campaigns

As noted, anti-stigma campaigns have largely been conceptualized using KABP paradigm (2, 4, 13). Furthermore, reflecting the agendas involved, mental health related stigma has predominately been considered to stem from the lack of professional knowledge. In this section, we will examine the three main ways public health professionals have challenged mental health related stigma: (2) education-based interventions; (3) protest-based interventions; and (4) contact-based interventions (3).

### The Knowledge-Attitude-Behavior Practice Paradigm

In line with a KABP paradigm, anti-stigma efforts have predominately been evaluated using the following questionnaires: the Mental Health Knowledge Scale (MAKS) (57); the Community Attitudes toward the Mentally Ill Scale (CAMI) (58); and the Reported & Intended Behavior Scale (RIBS) (59).

MAKS is split into two sections: one that evaluates how accurately the public recognizes psychiatric conditions, and another which evaluates how far the public agrees that professional help can support recovery (57). This is built on the prediction that the public's beliefs about the causes of mental health problems, as well their belief about whether someone with a mental health can fully recover, have a linear and singular relationship with an individual's levels of prejudice and discrimination (28, 60, 61). Prejudice is often evaluated using CAMI (58). These items cover attitudes about social exclusion, benevolence, tolerance, and support for community mental health care (58). Discrimination is predominately measured using a subsection of RIBS, which measures the public's willingness to live with, work with, live nearby, and continue a relationship with someone with a mental health problem (59). The other subsection of RIBS measures whether the public self-reports having had experienced each of these forms of contact (59).

Evaluations of anti-stigma campaigns come in three main forms. First, they compare the relative pre-test/post-test effectiveness of anti-stigma interventions in changing the public's knowledge, attitudes, and behaviors, as well as how these effects may vary by intervention type and target group [e.g., (7, 27, 28)]. Second, time trend studies, which have evaluated at a regional and national level, co-occurrences between exposure to public health campaigns and changes in mean responses [e.g., (16, 62)]. Third, cross-sectional or quasi-experimental techniques, which have measured the relationship between the content of education-based interventions and the contents of individual attitudes, behaviors, and affects [e.g., (63, 64)].

### Education-Based Interventions

Education-based interventions are the most common method used to challenge mental health-related stigma (6, 11, 13, 28). Reflecting a services agenda, these interventions have predominately, but not exclusively, relied on theories of MHL (8, 11, 28, 65). This is defined as “*the knowledge and beliefs about mental disorders, which aids their recognition, management or prevention*” [(66), p. 182]. Namely, advocates of MHL hope that providing the public with professional forms of knowledge will increase their engagement with professional services (7, 65).

Most interventions have been aimed at educating whole communities (67–71). There is evidence to suggest that these interventions may have small to medium positive effects in challenging stigmatizing forms of knowledge and attitudes (11, 28). This was the approach largely pursued in England in the first stages of the Time to Change Campaign (launched in 2008). Specifically, it aimed to target the whole English population via a large-scale mass media social marketing campaign, in which the public were presented with “myths” and “facts” about mental health problems (28). Similarly, this method was also used by the Beyond Blue campaign in Australia, although it placed a greater emphasis on encouraging the public to seek out professional help (72), engaging further with a services-agenda. Evaluations of both these campaigns have found a dose-effect relationship between exposure to the campaign and regional increases in MHL, positive attitudes toward professional forms of treatment, and help-seeking intentions (72, 73).

A focus on increasing the public's MHL is particuarly pronounced in low to middle-income countries (13), and simliar effects have been found have been found in these places (7, 11, 74, 75). Whilst, earlier reviewers pointed to common issues in low-evaluation follow up [e.g., (7, 13)], more recently researchers have noted there is a serious need to give greater consideration to the local contexts which situate understandings of mental health and illness (75, 76). Namely, around only 1 in 10 interventions have been developed “within” the country of intervention, and almost all interventions included some form of educational component (75).

Although cross-culturally we have seen an overall increase in the number of individuals who endorse “modern” understandings of the etiology of mental illness, concerns about trust in familial and work settings have been sustained (77, 78). Indeed, even in countries with high MHL, issues that deal primarily with intimate relationships (e.g., family), vulnerable groups (e.g., children), positions of authority, or power (e.g., work supervisors), or close forms of contact (e.g., shared accommodation), continue to elicit high negative responses (62, 78). It is the prohibitions on contact in these contexts (79) which are considered to be the “*backbone”* of stigma [(78), p. 853].

Reflecting a consideration that certain groups have a disproportionate role in challenging mental health related stigma, over the last 10 years education-based interventions have increasingly been targeted toward key groups (28). Key groups have often been identified on the basis of their frequency of contact with service users (e.g., health care professionals), their position of power (e.g., occupational and criminal services), or their potential for changing the future (students and young people) (3, 28, 80). However, very few have considered stigma at more than one level or the intersections between the multiple forms of health-related stigma (6, 81).

Reviews of mental-health related-stigma in health-care settings, suggest that education-based interventions can be effective in promoting positive attitudes about civil rights, especially for those with little or no formal mental health training (82) and may also reduce desires for social distance and increase feelings of empathy and self-efficacy (83). However, as studies have largely focused on attitudinal outcomes, knowledge, intentions and clinical competence (28), it is unclear how far these programs have challenged stigmatizing behaviors in practice.

Another trend has been the focus on MHFA (15). In many ways, MHFA could be considered an extended form of the traditional MHL programs (10, 84), with an added explicit risk framing (85). Namely, it promotes a belief that experiences of distress present a potential risk to the self and others (3, 86), and that this risk should be managed by promoting the public's ability to recognize the symptoms of distress and help individuals in distress receive professional services (7, 86). However, MHFA can be distinguished from these initial formulations of MHL by the importance it places on social networks (86). Recent reviews of MHFA suggest that it may be an effective method for increasing the public's MHL and intentions to seek to professional services (7), and it is hoped that these intentions will translate into real behavior (7, 87).

However, researchers have also expressed serious concerns about the possible unintended consequences campaigns may have (3, 4), although these effects are not often considered in a narrow application of the KABP paradigm (88). Of particular concern has been campaigns which have exclusively focused on increasing the public's biogenetic and neurological explanations of mental illness (4, 89, 90). This is problematic as both the diagnostic labeling of schizophrenia as an “illness” and biogenetic causal theories, are positively correlated with perceptions of dangerousness, unpredictability, fear, and desire for distance (17, 19). Moreover, the possible stigmatizing effect of genetic attributions may not be restricted to those with a form of mental health problem, as increases in genetic attributions are associated with an increased desire for social distance from the someone's sibling, particularly regarding intimate forms of contact such as dating, marriage, and having children (89). Furthermore, reviews largely find the endorsement of biogenetic causes to be associated with an increase in internalized stigma (18, 91) and may increase negative feelings of fear and guilt (63). Indeed, at the 3-month follow-up, an evaluation of the MHFA found the public to report being significantly less willing to tell someone that they have a mental health problem (92). Furthermore, it seems that the slight reduction in their belief that someone with a mental health problem is dangerous or unpredictable was replaced by a belief that they are weak (92). Whilst some researchers have suggested that biogenetic messages may be useful in motivating those with experiences of mental illness to take an active role in their treatment (18, 93), others have found it to reduce positive beliefs of recovery (94). In addition, it is important to note, that whilst on average among stakeholders, messages which emphasize the biogenetic and neurochemical causes of mental illness, and its treatability through medication, are highly unpopular, there is a clear divergence in opinion between psychiatrists and service-users (95).

We can also see these unintended effects at a national level. Meta-analyses of national time-trend studies found that whilst there was a trend toward greater MHL, in particular toward a biological model of mental illness and support for professional forms of treatment, there were also increases in desires for social distance from someone with a mental illness (19). For example, in the late 1990s the National Alliance on Mental Illness in the United States framed mental illness as a ‘brain disorder’ (3). Ten years later, although neurobiological explanations of depression and schizophrenia did increase, so too did desires for social distance and perceptions of dangerousness and unpredictability (96).

Recently, to limit these unintended effects, there has been some consideration of whether non-categorical messages are effective in challenging stigmatizing beliefs which divide between “us” and “them.” Whilst, there is reasonable correlational evidence to associate continuum-based messages with lower degrees of public and self-stigma (71, 97, 98), the evidence from experimental research is mixed (99). Specifically, whilst those participants exposed to continuum beliefs did see someone with experiences of schizophrenia as more similar to themselves and did increase their belief in possible recovery, the type of message did not significantly impact measures of explicit prejudice and discrimination (99). Similarly, an evaluation of the Time To Change campaign in the UK found that biopsychosocial messages relative to biomedical messages only had an effect on participants' desires for social distance in those who already understood mental illness in dimensional terms (100).

In addition, researchers need to be careful in using continuum-based belief interventions, as they may also have unintended consequences. Specifically, Thibodeau and Peterson (64) found continuum-belief interventions to increase participants experiences of anxiety and threat (64). This is concerning, as public health campaigns aimed at the public's perceptions of health-related threats, are also found to increase group-based prejudices, especially when the recommendation is perceived by the public to be outside of their control (101–103), a description often using by the public when making sense of someone with a mental health problem (21, 104).

In summary, whilst education-based interventions may have been productive in increasing the public's appetite for professional forms of intervention, their limited and likely unintended effects suggest that it may be time to retire their use as a method to challenge mental health related stigma (3, 40). Ultimately, however, reflecting practitioners' narrow use of the KABP paradigm, few interventions have explicitly considered possible unintended effects (88) limiting our ability to make firm causal statements.

### Contact-Based Interventions

In part in response to the limited and unintended effects education-based interventions have had, over the last 10 years there has been an appetite for interventions with elements of social contact.

Contact-based interventions are typically conducted in conjunction with an educational component (13, 65, 75), although they may also operate as stand-alone programs [e.g., (105–107)]. As the mechanisms involved in contact are poorly understood (28, 40, 108), public health professionals have often relied on a working definition of these programs, defining them as the “*interactions with people who have a mental illness to change prejudice”* [(28); p. 250].

In practice, contact-based interventions resist a singular definition, and have been used to describe an array of interventions. To illustrate this breadth, we will now briefly describe three national campaigns that all used some form of contact but differed notably in how they conceptualized and challenged mental health related stigma (109). The “Hjärnkoll” campaign in Sweden focused on creating activities and events to promote social contacts with people with lived experiences of mental illness (110). This came in four main forms: direct face-to-face contact in the community; mediated contact through the internet and media campaigns; contact through events organized by local charities; and discussion with managers in the workplace (110). Similarly, the second and third stages of the Time to Change Campaign in England have promoted indirect contact through a broad social marketing campaign including social media and the radio, and typically focused on portraying the friendships between young men (60). In contrast, the “Opening Minds” campaign in Canada did not include a mass media element (111). Rather it took a grass roots approach, in which individuals with experiences of mental illness shared their personal stories of recovery with those in their local community (111). Furthermore, the approach was highly targeted to focus on key groups, such as students, healthcare providers, the media, and human resource managers (111).

Contact-based interventions also vary notably in their understandings of expertise, reflecting the multiple agendas involved in challenging mental health related stigma. For example, in the “Hjärnkoll” campaign, those with experiences of mental illness were very much considered to be an expert by virtue of their experiences, and accordingly were referred to as “föreläsande ambassadörer” (lecturing ambassadors) (110), aligning closer with a self-worth agenda. In contrast, the “In Our Own Voice” campaign run by the National Association for Mental Illness (NAMI), places the emphasis on the expertise of mental health professionals (112). For example, in this campaign, service users undergo a 2-day training program where they learn to format their experiences to fit with the principles of MHL programmes (113, 114).

The evidence for contact-based interventions is mixed. Reviewers have typically concluded that contact-based interventions are more effective in challenging mental health related stigma than education-based interventions (3, 13, 28, 115) although not exclusively (11, 65, 116). After controlling for publication bias, contact-based interventions are considered to have small-to-medium effects in reducing stigmatizing attitudes and desires for social distance in the short term (7). However, it is questionable how far attitudinal changes and behavioral intentions are sustained after the intervention (7, 115, 117). Moreover, whilst population level surveys recurrently find having a close relationship with a person with a history of mental illness to be associated with less stigmatizing attitudes (28, 60, 110), a dose-relationship between contact-based interventions and stigma reduction is yet to be established (65). Specifically, reviewers have not found a relationship between the length or frequency of contact and the degree of stigma reduction (7, 117).

It is important to note that the evidence for contact-based interventions have largely come from comparisons between solely educational- and combined education-contact interventions (65). This is an important issue, as evaluations of stand-alone interventions have found mixed to no effects (9, 11, 116). Indeed, to date, almost all targeted interventions, such as those targeted the police services, have combined a mixture of education and contact-based interventions (28). Furthermore, it is important to remember that the groups that have been targeted for their potential to challenge mental health related stigma (e.g., mental health professionals) are also the most likely to have frequent, if not close, forms of contact (28), questioning how useful it is to consider individuals with experiences of mental illness as “unknown” or “unfamiliar” to these groups. Indeed, whilst some researchers did find a stronger effect of contact in mental health professionals (117), others have also found pessimistic beliefs about the reality and likelihood of recovery to be sustained (118), suggesting that researchers need to pay closer attention to processes involved.

To improve the effectiveness of interventions researchers have increasingly attempted to explicate the “active ingredients” involved in contact (20, 119, 120). To do, so they have often compared the relative effects of different forms of contact (7). The evidence regarding which form of contact is the most effective (e.g., face-to-face vs. video) is mixed and suggests there may be multiple relationships between type of contact, target audience, and form of mental health problem (7, 27, 115, 121). Furthermore, discussions with mental health professionals and service-users suggests that the content of interventions should be practical, encourage myth-busting, and emphasize recovery (20, 120). In addition, it may be useful to focus on engaging the public through shared activities and encouraging them to engage in anti-stigma advocacy efforts (119). However, there is by no means expert consensus (86), and thus far has only been validated in terms of attitudes not behaviors (118).

The lack of understanding about the casual mechanisms involved in contact-based interventions raises important questions about their continued utility. Indeed, as Gillespie (108) points out, a key continuance in the history of contact theory is the repeated discovery that contact is more complicated than we previously thought. Each discovery then encourages the development of an increasing list of conditions considered necessary for positive change. However, with each condition added, the theory is weakened, as it renders the theory impervious to falsification. Namely, failures to find an effect are explained not by the insufficiency of the theory, but instead, as a failure to fully operationalize the theory. Moreover, as the casual mechanisms of contact theory are poorly understood, it is hard to effectively apply the theory in real world situations.

Considering that most evaluations of stand-alone contact-based interventions found limited to mixed-effects (9, 11, 116), there is a dearth of research into contact without change. However, examining this occurrence reveals important aspects about how the public make sense of mental health and illness, and goes to the “backbone” of mental health related stigma (78). For example, Jodelet (122) documents a family colony in rural France in which patients from a local psychiatric hospital lived as “lodgers” in the homes of local families. At the time of the study, the program had been running for over 70 years, and it was common for multiple generations to grow up living with a lodger. However, despite the length and intimacy of the program, magical beliefs about madness were maintained, including fears of contamination. This was expressed through subtle ritualized forms of separation, such as an aversion to drinking from the same (washed) mug or handling liquid forms of medication. Whilst, the program would likely meet the criteria set for a contact-based intervention (e.g., sustained in-person contact with multiple individuals with a mental health problem) (111), stigmatizing beliefs about mental health problems were maintained.

In summary, it is clear that beliefs about contact are an important feature of the public's understandings of mental health and illness (13, 59, 122). Ultimately, it is possible that under certain conditions contact-based interventions may be a more effective method for challenging mental health related stigma than education-based interventions (3, 13, 28, 115). However, we currently lack the evidence base required to explicate the processes involved in why contact may, or may not, challenge mental health related stigma (65).

### Protest-Based Interventions

Although less common, national anti-stigma programs may also have conducted protest-based forms of intervention. Examples include the NAMI's StigmaBusters program (27) or SANE Australia's StigmaWatch program (112). These methods tend to align more closely with a rights-based agenda (3), and may consist of targeting stigmatizing advertisements, news stories, and forms of media entertainment through strategic letter-writing campaigns, press releases, marches, sit-ins and boycotts (9, 123). Furthermore, they may operate in conjunction with other education- and contact-based interventions (28, 110). However, whilst in theory protest-based methods challenge a broad array of injustices, in practice, their focus has mainly been on chastising the media for using psychiatric terminology out of context (112). Moreover, it has largely been a reactive strategy focusing on countering negative images about people with mental illness (123). This often includes calling out public bodies for promoting an understanding of mental illness in terms of unpredictability and violence (112), as well as those who sensationalize celebrity breakdowns (124).

As there have been few evaluations of protest-based interventions (9), the sample sizes are too small to be included in reviews comparing the effects of education- and contact-based interventions (27). However, some understanding of the effects of these campaigns may be gleaned from interventions targeted toward the media, although it is unclear how far these effects can be specifically attributed to protest-based methods (125). What is clear, is that there has been an overall reduction in the number of the news reports and social media posts which use psychiatric terminology out of context, and that this is more common for depression than schizophrenia (126, 127). However, it is questionable whether a reduction in content is a desired outcome, as public memories of news reports continue to prioritize images of violence and have not been associated with a reduction in desire for social distance (128). Indeed, it seems that protest-based methods may have reduced the overall amount of content about mental illness, rather than changed publics beliefs or behaviors.

## How The Public Make Sense of Mental Health And Illness

It may be important for public health professionals to reconsider how KABP paradigm responds to the ways the public make sense of mental health and illness. Whilst, linear and individualistic models of behavior change are appealing for their simplicity, and the clear policy responses they suggest, once context is taken into account, they often fail to appreciate how health-related behaviors are embedded in the flow of everyday social practices (129, 130). These are typically conducted without self-conscious reflection, and instead rely upon practical or tacit knowledge, that which is often treated as “common-sense” (2, 129).

As previously discussed, public stigma describes “*the contextual climate of prejudice and discrimination*” [(45), p. 94]. Examinations of this contextual climate have consistently found group-based practices that Other individuals with experiences of mental ill-health. Broadly, Kalampalikis and Haas (131) define the Other as a belief that guarantees, orchestrates, or institutes difference, something that may often involve descriptions of being uncommon, non-familiar, strange and fundamentally “not-me.” Cross-culturally, this treatment ranges from its media portrayal to the beliefs expressed in professional and lay communities (76, 104, 122, 132–134).

Furthermore, it is important to note that this contextual climate is both structured and contested (3, 135, 136). Namely, there is limited consensus both over the “nature” of mental illness or how to challenge its stigmatization (95). Whilst the services-agenda has somewhat singularly focused on remediating the public's perceived lack of professional knowledge, those who advocate a rights-based agenda often emphasize the asymmetric power relations that connect stigmatizing attitudes and beliefs with discriminatory consequences (44, 51).

Appreciating the contested nature of mental health related stigma has profound implications for the continued utility of attitude-based research, a principal component of intervention design and evaluation (8). Specifically, this suggests that understandings of mental health and illness are a feature of public life, and that in times of contestation, individuals and groups are required to advance their particular forms of understanding (135, 136). This is in contrast to attitude-based theories, which often assume individuals to be agnostic toward their attitudes (137). Indeed, even when researchers have attempted to contextualize or structurally locate individual attitudes [e.g., (34, 138)], they often overlooked the power struggles involved in developing public consensus (129, 139). This is of serious concern, as doing so obscures the asymmetric power relationships involved in defining what is taken for granted (139). It is these notions which have been shown to allow the public to think, feel and behave toward someone they perceive to have a mental health problem (21, 122, 136).

This has led some researchers to argue that it may be more productive to consider what particular groups have at stake in maintaining their particular understandings of mental health and illness (8, 51, 140). Indeed, it may be useful to consider individual attitudes as a motivated form of cognition, whose expression provides insight into lay concepts of the social order (63, 141). However, the social order cannot be fully reduced to the explicit contents of individual attitudes. Rather, especially when close attention is paid to the contexts considered to be the “*backbone”* of mental health related stigma (78), common-sense thinking about mental health and illness are expressed through a wide constellation of contextually-defined affects, rituals, images, narratives, and gestures (122, 132, 142), whose meanings are often embodied in the process of everyday life (21, 122, 143).

These constellations of meaning should be considered motivated. Even during sustained interaction, the public are frequently found to represent mental health problems as existing in different spaces and times (36, 144, 145). This often involves describing someone with a mental health problem as distant, foreign, or “out-there” (21, 36, 146). Moreover, these metaphors reflect beliefs held about the spaces thought to locate mental illness, namely the psychiatric asylum, a space which prioritizes beliefs of violence, loss, and contamination (36, 134, 144, 145, 147). Similarly, examinations of public understanding recurrently find that the public place prohibitions against sharing intimate objects (e.g., door knobs, drinking cups, toilet seats), and that the violation of these prohibitions is found to elicit distance-promoting affects of fear and disgust (21, 79, 122).

Indeed, the close examination of these contexts calls into question the very utility of a KABP paradigm. Namely, in contrast to the key assumption that public knowledge is singular, once context is taken into account, the public are found to be polyphasic in their understandings of mental illness (148, 149). Cognitive polyphasia refers to the dynamic co-existence of multiple distinct modalities of knowledge rooted in the multiple relationships between individuals and their environments (136, 150). This is expressed in two ways. First, practices which Other mental illness involve often multiple beliefs. This ranges from beliefs of contagion and demonic possession to more “modern” biomedical forms of knowledge (21, 104, 122, 148, 149). Second, differentiated forms of understanding between types of mental illness do not necessarily disrupt the mental illness degenerated position in the social order (140). Namely, whilst schizophrenia is recurrently found to elicit more negative attitudes and beliefs than depression (78, 104), this is does not overcome the strength of mental illness's unified representation as Other (21, 140).

Drawing on their common-sense notions, individuals and groups intersubjectively sustain and challenge understandings of mental health and illness (136). From an intersubjective perspective, “*not only must the other be physically present with its own body, but the other must also recognize the subject as an intentional and self-conscious self”* [(151), p. 1]. Whilst the nature of this engagement is culturally defined (51), it always involves a transaction between the Self, the object of consideration (i.e., mental ill-health), and a social Other (e.g., family member, friend, mental health professional) (152). This is of key importance as, rather than mental illness being fully “unknown” or “incomprehensible” to the public, beliefs about mental health and illness constitute an important form of self-knowledge. For example, the public often refer to personal experiences when asked to explain their beliefs about mental health and illness (76, 104, 122). Furthermore, whilst the public often legitimize fears of contact by contrasting the perceived unfamiliarity of schizophrenia with the perceived familiarity of depression (19, 104), population-level surveys suggest that up to three-quarters of the public have at some point experienced psychotic-like experiences (153, 154). Indeed, rather than mental illness being fundamentally “unknown,” evidence suggests it may be in part the public's recognition of experiences of distress, that motivates them to sustain distancing affects, beliefs, and behaviors (21, 36, 64, 122, 140).

## Why Might the Public Resist Anti-stigma Efforts?

In line with service-based understandings of mental health-related stigma, over the last 20 years public health professionals have increased the public's biomedical explanations of mental illness. However, mental illness remains Othered; a practice which often involves prohibitions around close forms of contact (21, 78, 79, 122). In this section, we will elucidate the psychological mechanisms that may explain why these campaigns have had limited- and mixed effects.

To review, in contrast to the assumptions made in KABP paradigm, public understandings of mental health and illness are often not singular. Rather the public are found to maintain polyphasic understandings of mental health and illness, although these multiple forms of understanding are often expressed outside of conscious awareness. In particular, they often expressed through affectively-laden prohibitions on close forms of contact (21, 51, 122), the content of which expresses localized cultural beliefs about the social order (51, 141). Additionally, rather than these understandings being held in the “abstract,” they are both motivated and constitute an important form of self-understanding (4, 21, 36).

Examinations of public understanding find Othering to be an important mechanism in sustaining mental health related stigma. Specifically, at the level of representation, the public are found to dissociate themselves from groups they see as Other (23, 155). Indeed, the historical record suggests Othering may be an effective method for the public to distance themselves from threats seen as contagious, foreign or unknown (e.g., HIV/AIDS) (23, 25, 79, 156). For example, it is well-established that media representations of mental illness frequently prioritize representations of violence and despair (36, 134), a representation the public are found to respond to both through beliefs of psychological difference and distancing-maintaining behaviors (21, 51). Similarly, a more recent manifestation of Othering is the belief that the public would not know how to interact with someone with a mental health problem (157, 158), despite mental health and illness being very much an important form of self-knowledge (36).

The tenacity of Othering mental health problems may in part be explained by distancing-blame-stigma patterns, a common response to health-related threats (23, 155). Namely, to maintain beliefs of difference between “us” and “them,” the public are recurrently found to emphasize aspects considered to render the afflicted disproportionately susceptible to the perceived threat (23). One manifestation of this is the public's continued appetite for biogenetic and neurological explanations of mental illness over those that which encourage the public to see someone with experiences of distress as a whole person (3, 95). Whilst this is not to suggest that mental illness has no genetic and neurological basis, it is important to note these explanations can be highly effective at maintaining a perception that neither I, nor my in-group, will experience some form of psychological distress (36).

These inter-group practices are often valanced to include negative out-group attributions of responsibility and blame (23, 158, 159). Indeed, a common finding is that marginalized or derogated groups are imagined to be both uniquely susceptible to illness and responsible for their misfortune (23, 81), especially when the illness is considered to be caused by unknown or multiple causes (156). To note, whilst much of the literature on these distance-blame-stigma patterns has come from interventions to limit HIV/AIDS, a recent focus on intersectionality has highlighted that both HIV/AIDS and mental health related stigma at their core are about inequalities in the social order (81).

Whilst it was hoped that emphasizing the public's biomedical knowledge would displace the public's long held belief that individuals with experiences of mental illness are “bad” (4, 104), it seems that polyphasic forms of understanding have been sustained (136). Namely, by promoting a belief that the actions of individuals with a mental health problem are rooted in their genetics or neurology, and hence potentially considered beyond conscious awareness, existing concerns about unpredictability and dangerousness were sustained (90, 160). Furthermore, these perceived risks are likely to have been exacerbated, as increases in biogenetic and neurological explanations of mental illness are consistently correlated with a belief that mental illness is unrecoverable (17–19). Additionally, examinations suggest that rather than displacing the perceived Otherness of mental illness, biomedical explanations of mental illness are frequently drawn upon by the public to legitimize their relative fears of perceived groups of mental illness (e.g., Psychotic vs. Mood disorders) (21, 140). This practice maintains a unified representation of mental illness as Other (23, 140).

As noted, examinations reveal public understandings of mental illness to be motivated and involve aspects of self-knowledge. Specifically, to protect the Self from the perceived threat posed by mental illness, the public are found to engage strategies that maintain a representation of mental illness as “not-me.” This representation is arguably pacifying. Namely, it helps protect the Self from what is often feared: experiences of mental ill-health (21, 26, 36, 140, 161). Indeed, since antiquity, mental disturbances have been represented as having profoundly disruptive effects, both for those experiencing the illness and for those around them (161). Moreover, as the public often consider mental illness to involve disorders of perception, volition and morality, experiences of mental illness are considered to threaten the very experience of living (161). Indeed, one could consider Othering to be a highly functional, but unjustifiable, social practice, as it affords the public psychological protection (21, 26, 141).

As described, through the MHFA, practitioners have increasingly framed mental illness in terms of risk, both to the self and to others. Whilst, we are not arguing that in certain circumstances individuals with experiences of mental ill-health may need access to extra services and protections, using risk framings as a method to challenge public stigma is highly problematic (101). Specifically, a recurrent theme in the literature on health and stigma, is that collective practices which attribute risk to a particular group (i.e., individuals with mental illness) often is concomitant with discursive practices that Other the afflicted group (25, 162, 163). Indeed, groups which are constructed by the lay public as “at-risk,” are also often materially and symbolically believed to threaten the social order (25, 162). In addition, these constructions are often concomitant with discriminatory practices that unjustifiably remove marginalized groups from public life (79, 164). For example, in the British context, we can see this in the media discourse surrounding the 2002 Mental Health Bill. Whilst a wide number of interested organizations, ranging from the Royal College of Psychiatrists and the Law Society, to the Mental Health Alliance, all considered the bill to be overly focused on the notion of the perceived threat posed to the general public at the expense of service-user rights and freedom, reporting on the bill implicitly sustained a belief that the public need to restrict the movements of service-users before they can a pose a perceived threat (135).

Whilst a diametric opposition between the Self and Other is remarkably historically and cross-culturally consistent (23), the content involved is always particular to the context in which it is practiced (141, 165). For example, in the Chinese context, mental illness is considered as a form of social death considered to threaten the moral and material value of the family (51, 166). In contrast, in the western context, where a greater degree of value is placed on in individual choice and self-reliance, individual's with experiences of mental illness are often degenerated as lacking rationality and self-control (21, 26).

In addition, as these sense making processes are rooted in the everyday task of living, it is important to pay serious consideration to the structural influences which locate understandings of mental health and illness (136). For example, in the Indian context, it has been found that women living in low-income settings, who have an increased likelihood of experiencing gender-based violence, understood the psychological and behavioral experience of distress in terms of family relationships, social roles and poverty, themes also considered to cause mental illness or “madness” (76). However, despite their shared causes, mental illness remained Othered, with participants considering someone with experiences of “madness” to be qualitatively different, a representation achieved through beliefs of danger, difference and more extreme social consequences (76). Similarly, in the British context, where the likelihood of experiencing mental ill-health is structured by socio-economic status, groups who have increased levels of familiarity through personal experience, are also more likely to consider mental illness as unfamiliar (167), suggesting mental illness is distanced at the level of representation. In addition, those most in need of anti-stigma efforts are suggested to be even more likely to develop knowledge about mental illness through their interactions with services, and hence be less responsive to fully informational based campaigns (167).

## Future Directions for Research

We have evidenced that interventions have relied on an insufficient conceptualization of mental health-related stigma. Specifically, whilst applications of the KABP paradigm have assumed mental health and illness to be “unknown” or “unfamiliar” to the public, at the level of representation, the public continue to engage in strategies which Other individuals with experiences of mental ill-health, even in groups with high MHL and high frequencies of contact (28, 78). Furthermore, we need to heed the unintended consequences campaigns have had in maintaining beliefs of difference between “us” vs. “them,” especially those with have exclusively focused on educating the public in biogenetic and neurochemical explanations of mental illness. In response, in this section we take inspiration from the broader behavior change literature, and suggest how practitioners might develop new interventions rooted in the multiple ways the public make sense of mental health and illness.

Whilst, practitioners working in the broader health promotion domain have recurrently reflected on the need to develop new interventions which appreciate the complexity of social life, the field continues to focus on individualized explanations of behavior change (168), often resulting in limited and mixed effects (129, 169). In response, some practitioners are starting to argue that it may be more productive to focus on the context and variability of health-related behaviors, rather than a focus on programmatic or unified theories of change (129, 169).

To do so, one method that is increasingly being explored is “interweaving” (170). This refers to approaches which select the particular context of intervention at the start of the research process (171). In some ways, this fits practitioner's current focus on targeting key groups groups. However, this goes further, as interweaving requires a full examination of the particular physical, cultural, economic, and political architectures which locate sense-making about mental health and illness before intervention (168, 170). Indeed, doing so responds to a key inadequacy of the KABP paradigm—that knowledge is only considered in its abstract form (4)—and instead promotes a contextualized understanding of mental health-related knowledges as embodied and functional (172).

In exploring the contexts of public understanding, we encourage practitioners to pay attention to three principles. First, they should locate individual behavior in the physical, social and organizational environments in which they take place (173). This is important, as both the content and process of Othering are culturally and structurally determined. Second, a broad array of stakeholders should be fully engaged throughout research process (3, 4, 171, 173). In particular, to ensure empowerment remains a key objective of anti-stigma campaigns (40, 173), the voices of those with lived experiences of distress should be centered throughout the research process (3, 51). Third, as causal explanations of change often require interdisciplinary research and engagement of both clincial and non-clinical researchers (30, 174, 175), consideration should be given to multiple theories of mental health related stigma (3). Consequently, an iterative approach to intervention design and evaluation should be taken (30). This will likely require a triangulation of qualitative and quantitative methodologies, which may be conducted sequentially or in-parallel (176).

By paying closer attention to context, hopefully insight will be provided into the limited and mixed-effects contact-based interventions have had (9, 11, 116). Indeed, a focus on how individuals and groups develop representations in and through contact, arguably turns our conceptualization of contact-based interventions on its head. Rather than assuming experiences of mental ill-health to be “unknown” or “unfamiliar” to the public, a focus on process exposes the socio-environmental causes which determine whether mental health and illness is perceived to be a feature of everyday life (108). That is, by centring context in the analysis, researchers can consider how groups even in close physical context maintain a representation of mental illness as “foreign,” “different,” and fundamentally “not-me” (131). In selecting these contexts, practitioners should pay particular attention to those which involve intimate relationships, differentials in power and perceived vulnerable groups, as it is these forms of contact which are found to be central to mental health related stigma (78).

In addition, as Othering is achieved in part at the level of representation, researchers should not reduce the research field to a wholly material understanding of space. Rather, in line with recent examinations of “more-than-human” spaces (177), researchers should consider the idiographic aspects of representation, and examine how groups may implicitly draw on spatialized representations (e.g., the asylum as foreign) to maintain personal positive forms of social identity and degenerate those with experiences of mental ill-health (144). Furthermore, there is a serious need to closely examine what is taken-for-granted in these spaces, as this form of knowledge provides important insight into the discursive and material practices which sustain a representation of mental illness as Other (143). For example, individuals with mental illness have been found to be represented in strange and chaotic spaces and are less likely to be portrayed in everyday situations (132). To do so, it may be productive for researchers to relegate professionalized models of mental health related stigma, and instead take a subjugated position toward their participants, as this often allows them to express multiple forms of knowledge, which may or may not fit within professionalized paradigms (133), and is a technique which has been productively used to examine the knowledges practiced by both service-users and mental health professionals (133, 178).

Moving forward, practitioners may find it useful to also consider interventions conducted outside of the health-related stigma domain. In particular, as mental health related stigma is sustained through ritualized prohibitions on close shared forms of contact, often practiced outside of conscious awareness, it may useful for learn from practitioners who intervened in the physical environment to limit collective practices in Othering. For example, Joffe et al. (179) designed a “Fix-it” intervention to increase the publics earthquake and home fire preparedness. Although there had been a number of national campaigns (e.g., American Red Cross Home Fire Preparedness Campaign) focused on increasing awareness of potential hazards, there was limited evidence for sustained behavioral change (179), and common cross-cultural practices in Othering were sustained (180). To respond to this, researchers developed a program in which participants took part in two 3-h interactive face-to-face workshops focused on securing items in the household. Rather than increasing the publics knowledge about the causes and effects of natural hazards, the fix-it intervention focused on practical changes that could be made to the physical environment. An aspect of which involved taking photographs of secured objects in their own home. Furthermore, as Othering is both collective and individual, participants were invited to share their learning on a fix-it Facebook group page designed and manged by one the researchers.

A cross-cultural evaluation of the intervention highlights the need to consider routinised individual behaviors within the wider social environment (181). Specifically, collective efficacy—the perception of one's community's ability to prepare for a hazard—had a greater on individual preparedness in Turkey relative the USA, where a greater emphasis is placed impact upon individual efficacy (181). In addition, highlighting the need to contextualize individual behavior in the socio-political environment, they suggest robust legislation sets important social norms for behavior and locating individual notions of responsibility (181).

It is our intention that this inspires practitioners to action and for them to develop new interventions rooted in the multiple ways the public make sense of mental health and illness. Whilst Othering as a motivated and collective practice is very much in evidence, reflecting the near exclusive use of a KABP paradigm, the main criticism we can make of the evidence we have presented, is that we have drawn on broadly descriptive literature, rather than one developed directly thorough anti-stigma interventions. Indeed, there is both a serious lack of interventions which have explicitly been designed to target these distance-blame-stigma practices and the necessary measures needed to evaluate them.

## Conclusion

Given the significant literature associating biomedical explanations of mental illness with public desires for social distance, there is serious reason to contend that education-based interventions, especially those which have exclusively focused on biomedical explanations of mental illness, have sustained public practices which Other and distance those with experiences of mental illness. Additionally, whilst research shows notions of contact are central to mental health related stigma, we lack causal evidence for contact-based interventions. Indeed, it is especially questionable whether mental illness can be considered “unknown” or “unfamiliar” to those most in need of anti-stigma efforts.

It is for these reasons that we contend there is a serious need for new interventions to be developed rooted in the ways the public make sense of mental health and illness. To some extent, public health campaigns are moving in this direction through the establishment of the Global Anti-Stigma Alliance (GASA) (182), a group of 20 members who conduct national anti-stigma campaigns in Western Europe, North America, and Australasia (e.g., SANE Australia, Time to Change England, & Opening Minds Canada). For example, GASA outlines that anti-stigma campaigns should focus on empowering those with lived experiences of mental health problems to design and lead grassroots social movements (182). Furthermore, they argue anti-stigma programs should focus on promoting the dignity and rights of those who have experienced stigma and discrimination (182). However, it is important to remember that these programmes do not fully operate independently to those pursuing a services agenda (183).

In summary, this review evidences the need for a paradigmatic shift away from a KABP paradigm to a contextualized understanding of the processes which sustain mental health related stigma. It is our hope, that by examining if, how and why, even in close forms of contact, the public sustain practices which represent individuals with experiences of mental illness as Other, that in 20 years' time we can consider antistigma efforts in terms of success rather than failure.

## Author Contributions

DW and JF contributed to all stages of the manuscript, including conceptualization, drafting, and editing. Both authors contributed to the article and approved the submitted version.

## Conflict of Interest

The authors declare that the research was conducted in the absence of any commercial or financial relationships that could be construed as a potential conflict of interest.

## References

[1] World Health Organization Mental Health: New Understanding, New Hope. Geneva: World Health Organization (2001)

[2] Joffe H. AIDS research and prevention: a social representational approach. Br J. Med Psychol. (1996) 69:169–90. 10.1111/j.2044-8341.1996.tb01863.x 8883972

[3] Corrigan P The Stigma Effect: Unintended Consequences of Mental Health Campaigns. New York, NY: Columbia University Press (2018). 10.7312/corr18356

[4] Foster J Mental health campaigns and social representations theory: a consideration. Papers Soc Rep. (2017) 26:4.1–21. 10.17863/CAM.28049

[5] Henderson C Gronholm P. Mental health related stigma as a 'wicked problem': the need to address stigma and consider the consequences. Int J Environ Res Public Health. (2018)15:1158. 10.3390/ijerph15061158 29865225 PMC6024896

[6] Rao DE. A systematic review of multi-level stigma interventions: state of the science and future directions. BMC Med. (2019) 17:41. 10.1186/s12916-018-1244-y 30770756 PMC6377735

[7] Morgan A Ross A Reavley N. Systematic review and meta-analysis of mental health first aid training: effects of knowledge, stigma, helping behavior. PLoS ONE. (2018) 13:e0197102. 10.1371/journal.pone.0197102 29851974 PMC5979014

[8] Angermeyer M Schomerus G. State of the art of population-based attitude research on mental health: a systematic review. Epidemiol Psychiatr Serv. (2017) 26:252–64. 10.1017/S2045796016000627 27573679 PMC6998641

[9] Griffiths K Carron-Arthur B Parsons A Reid R. Effectiveness of programs for reducing the stigma associated with mental disorders. a meta-analysis of randomized controlled trials. World Psychiatry. (2014) 13:161–75. 10.1002/wps.20129 24890069 PMC4102289

[10] Jorm A. Mental health literacy: empowering the community to take action for better mental health. Am Psychol. (2012) 67:231–43. 10.1037/a0025957 22040221

[11] Mehta N Clement S Marcus E Stona AC Bezborodovs N Evans-Lacko S. Evidence for effective interventions to reduce mental health-related stigma and discrimination in the medium and long term: systematic review. Br J Psychiatry. (2015) 207:377–84. 10.1192/bjp.bp.114.151944 26527664 PMC4629070

[12] Corrigan P Watson A. Understanding the impact of stigma on people with mental illness. World Psychiatry. (2002) 1:16–20. 16946807 PMC1489832

[13] Thornicroft G Mehta N Clement S Evans-Lacko S Doherty M Rose D Evidence for effective interventions to reduce mental-health-related stigma and discrimination. Lancet. (2016) 12:1123–32. 10.1016/S0140-6736(15)00298-6 26410341

[14] Robinson EH. Public knowledge, attitudes, social distance and reporting contact with people with mental illness 2009-2017. Psychol Med. (2019) 49:2717–26. 10.1017/S0033291718003677 30569883

[15] Morgan A Reavley N Ross A Too L Jorm A. Interventions to reduce stigma towards people with severe mental illness: systematic review and meta-analysis. J Psychiatr Res. (2018) 103:120–33. 10.1016/j.jpsychires.2018.05.017 29843003

[16] Angermeyer M Holzinger A Carta M Schomerus G. Biogenetic explanations and public acceptance of mental illness: systematic review of population studies. Br J Psychiatry. (2011) 199:367–72. 10.1192/bjp.bp.110.085563 22045945

[17] Loughman A Haslam N. Neuroscientific explanations and the stigma of mental disorder: a meta-analytic study. Cog Res. (2018) 3:43. 10.1186/s41235-018-0136-1 30426319 PMC6234201

[18] Larkings J Brown P. Do biogenetic causal beliefs reduce mental illness stigma in people with mental illness and in mental health professionals? A systematic review. Int J Mental Health Nursing. (2017) 27:928–41. 10.1111/inm.12390 28942615

[19] Schomerus G Schwahn C Holzinger A Corrigan P Grabe H Carta M. Evolution of public attitudes about mental illness: a systematic review and meta-analysis. Acta Psychiatr Scand. (2012) 125:440–52. 10.1111/j.1600-0447.2012.01826.x 22242976

[20] Corrigan P Vega E Larson J Michaels P McClintock G Krzyzanowki R The california schedule of key ingredients for contact-based antistigma programs. Psychiartic Rehabil J. (2013) 36:173–9. 10.1037/prj0000006 23834612

[21] Walsh DF. A contagious other? Exploring the public's appraisals of contact with 'mental illness'. Int J Environ Res Public Health. (2020) 17:2005. 10.3390/ijerph17062005 32197437 PMC7142778

[22] Fominaya AC. The effects of pity on self- and other-perceptions of mental illness. Psychiatry Res. (2016) 241:159–64. 10.1016/j.psychres.2016.04.058 27175911

[23] Joffe H. Public apprehension of emerging infectious diseases: are changes afoot? Public Understand Sci. (2011) 20:446–60. 10.1177/0963662510391604 21936260

[24] Eicher V Bangerter A. Social representations of infectious diseases. In: Gordon S Andreouli E Gaskell G Valsiner J editors. The Cambridge Handbook of Social Representations. Cambridge: Cambridge University Press (2015)

[25] Joffe H Risk and 'The Other'. Cambridge: Cambridge University Press (1999). 10.1017/CBO9780511489846

[26] Joffe H Staerkle C The centrality of the self-control ethos in western aspersions regarding outgroups: a social representational approach to stereotype content. Culture Psychol. (2007) 13:395–418. 10.1177/1354067X07082750

[27] Corrigan P Morris S Michaels P Rafacz J Rusch N Challenging the public stigma of mental illness: a meta-analysis of outcome studies. Psychiatr Serv. (2012) 63:963–73. 10.1176/appi.ps.201100529 23032675

[28] Gronholm P Henderson C Deb T Thornicroft G. Interventions to reduce discrimination and stigma: the state of the art. Soc Psychiatr Psychiatr Epidemiol. (2017) 52:249–58. 10.1007/s00127-017-1341-9 28144713 PMC5344948

[29] Mitroff I Featherlingham T On systematic problem solving and the error of the third kind. Behav Sci. (1974) 19:383–93. 10.1002/bs.3830190605

[30] Ruiter R Crutzen R de Leeuw E Kok G Changing behavior using theories at the interpersonal, organisational, community, societal levels. In: Hagger M Cameron L Hamilton K Hankonen N Lintunen T editors. The Handbook of Behavior Change. Cambridge: Cambridge University Press (2020). p. 251–66. 10.1017/9781108677318.018

[31] Clavier C de Leeuw E Health Promotion and the Policy Process. Oxford: Oxford University Press (2013). 10.1093/acprof:oso/9780199658039.001.0001

[32] Gelius P Rutten A. Conceptualizing structural change in health promotion: why we still need to know more about theory. Health Promot Int. (2018) 33:657–64. 10.1093/heapro/dax006 28334852

[33] Lieberman L Golden S Earp J. Structural approaches to health promotion: what do we need to know about policy and environmental change? Health Educ Behav. (2013) 40:520–5. 10.1177/1090198113503342 24048612

[34] Rose N Inventing our Selves: Psychology, Power, and Personhood. Cambridge: Cambridge University Press (2010)

[35] Crossley N Contesting Psychiatry. Social Movements in Mental Health. Abingdon, VA: Routledge, Taylor and Francis Group (2006). 10.4324/9780203000878

[36] Harpin A Madness, Art, and Society. Abingdon, VA: Routledge (2018). 10.4324/9781315149257

[37] Kutchins H Kirk S. Making Us Crazy. DSM: The Psychiatric Bible and the Creation of Mental Disorders. New York, NY: The Free Press (1997)

[38] Scull A Madness in Civilization. London: Thames & Hudson Ltd (2020)

[39] Schulz B. Stigma and mental health professionals: a review of the evidence on an intricate relationship. Int Rev Psychiatry. (2007) 19:137–55. 10.1080/09540260701278929 17464792

[40] Stuart H Arboleda-Florez J Sartorius N Paradigms Lost: Fighting Stigma and the Lessons Learned. Oxford: Oxford University Press (2012). 10.1093/med/9780199797639.001.0001

[41] Charles J Bentley K. Measuring mental health provider-based stigma: development and initial psychometric testing of a self-assessment instrument. Commun Mental Health J. (2017) 54:33–48. 10.1007/s10597-017-0137-4 28378302

[42] Kopera M Suszek H Myszka M Gmaj B Ilgen M Wojnar M. Evaluating explicit and implicit stigma of mental illness in mental health professionals and medical students. Commun Mental Health J. (2015) 51:628–34. 10.1007/s10597-014-9796-6 25535045 PMC4475542

[43] Stull L McGrew J Salyers M Ashburn-Nardo L. Implicit and explicit stigma of mental illness: attitudes in an evidence-based practice. J Nervous Mental Dis. (2013) 201:1072–9. 10.1097/NMD.0000000000000056 24284643 PMC4031039

[44] Link B Phelan J Conceptualizing stigma. Annu Rev Sociol. (2001) 27:363–85. 10.1146/annurev.soc.27.1.363

[45] Pescosolido B Martin J. The stigma complex. Annu Rev Soc. (2015) 41:87–116. 10.1146/annurev-soc-071312-145702 26855471 PMC4737963

[46] Hatzenbuehler M. Structural stigma and health inequalities: research evidence and implications for psychological science. Am Psychol. (2017) 71:742–51. 10.1037/amp0000068 27977256 PMC5172391

[47] Clement S Schauman O Graham T Maggioni F Evans-Lacko S Bezborodovs N. What is the impact of mental health-related stigma on help-seeking? A systematic review of quantitative and qualitative studies. Psychol Med. (2015) 45:11–27. 10.1017/S0033291714000129 24569086

[48] Allport G The Nature of Prejudice. Garden City, NY: Doubleday (1954).

[49] Bracken P Thomas P Timimi S Asen E Behr G Buester C. Psychiatry beyond the current paradigm. Br J Psychiatry. (2012) 201:430–4. 10.1192/bjp.bp.112.109447 23209088

[50] Middleton H Moncrieff J Crtical psychiatry: a brief overview. BJPsych Adv. (2019) 25:47–54. 10.1192/bja.2018.38

[51] Yang L Kleinman A Link B Phelan J. Culture and stigma: adding moral experience to stigma theory. Soc Sci Med. (2007) 64:1524–35. 10.1016/j.socscimed.2006.11.013 17188411

[52] Goffman E Stigma: Notes on the Management of Spoiled Identity. New York, NY: Prentice-Hall Inc (1963).

[53] Muntaner C Eaton W Diala C Socioeconomic inequalities in mental health:A review of concepts and underlying assumptions. Health. (2000) 4:89–15. 10.1177/136345930000400105

[54] Quinn D Chaudoir S. Living with a conceable stigmatized identity: the impact of anticipated stigma, centrality, salience, and cultural stigma on psychological distress and health. J Personal Soc Psychol. (2009) 97:634–51. 10.1037/a0015815 19785483 PMC4511710

[55] Foster J Reflections on bauer and gaskell's towards a paradigm for research on social representations. Papers Soc Rep. (2011) 20:1–12.

[56] Morant N. Social representations and professional knowledge: the representation of mental illness among mental health practitioners. Br J Soc Psychol. (2006) 45:817–38. 10.1348/014466605x81036 17393882

[57] Evans-Lacko S Little K Meltzer H Rose D Rhydderch D Henderson C Development and psychometric properties of the mental health knowledge schedule. Can J Psychiatry. (2010) 55:440–8. 10.1177/070674371005500707 20704771

[58] Taylor M Michael D. Scaling community attitudes toward the mentally ill. Schizophr Bull. (1981) 72:225–40. 10.1093/schbul/7.2.225 7280561

[59] Evans-Lacko S Rose D Little K Flach C Rhydderch D Henderson C. Development and psychometric properties of the reported and intended behavior scale (RIBS): a stigma-related behavior measure. Epidemiol Psychiatr Sci. (2011) 20:263–71. 10.1017/S2045796011000308 21922969

[60] Gonzalez-Sanguino C Potts L Milenova M Henderson C. Time to change's social marketing campaign for a new target population: results from 2017 to 2019. BMC Psychiatry. (2019) 19:417. 10.1186/s12888-019-2415-x 31881957 PMC6933720

[61] Pescosolido B Martin J Lang A Olafsdottir S. Rethinking theoretical approaches to stigma: a framework integrating normative influences on stigma (FINIS). Soc Sci Med. (2008) 67:431–40. 10.1016/j.socscimed.2008.03.018 18436358 PMC2587424

[62] Henderson C Potts L Robinson E. Mental illness stigma after a decade of time to change England: inequaliteis as targets for further improvement. Eur J Public Health. (2020) 30:497–503. 10.1093/eurpub/ckaa013 32531039 PMC7292343

[63] Rusch N Corrigan P Todd A Bodenhausen G. Implicit self-stigma in people with mental illness. J Nervous Mental Disord. (2010) 198:150–3. 10.1097/NMD.0b013e3181cc43b5 20145491

[64] Thibodeau R Peterson K. On continuum beliefs and psychiatric stigma: similarity to a person with schizophrenia can feel too close for comfort. Psychiatr Res. (2018) 270:731–7. 10.1016/j.psychres.2018.10.070 30551317

[65] Jorm A. Effect of contact-based interventions on stigma and discrimination: a critical examination of the evidence. Psychiatr Serv. (2020) 71:735–77. 10.1176/appi.ps.201900587 32188364

[66] Jorm A Korten A Jacomb P Christensen H Rodgers B Pollitt P. “Mental health literacy”: a survey of the public's ability to recognize mental disorders and their beliefs about the effectiveness of treatment. Med J Austr. (1997) 166:182–6. 10.5694/j.1326-5377.1997.tb140071.x 9066546

[67] Luty J Rao H Arokiadass S Easow J Sarkhel The repentant sinner: methods to reduce stigmatized attitudes towards mental illness. Psychiatrist. (2008) 32:327–32. 10.1192/pb.bp.107.018457

[68] Kimmerle J Cress U The effects of TV and film exposure on knowledge about and attitudes toward mental disorders. J Commun Psychol. (2013) 41:931–43. 10.1002/jcop.21581

[69] McGinty E Goldman H Pescosolido B Barry C. Portraying mental illness and drug addiction as treatable health conditions: effects of a randomized experiment on stigma and discrimination. Soc Sci Med. (2015) 126:73–85. 10.1016/j.socscimed.2014.12.010 25528557

[70] Schlier B Lange P Weise S Wirth A Lincoln T. The effect of educational information about treatments for schizophrenia on stigmatizing perceptions. J Behav Ther Exp Psychiatry. (2016) 52:11–6. 10.1016/j.jbtep.2016.02.002 26949924

[71] Wiesjahn M Jung E Dremser J Winfried R Lincoln T. The potential of continuum versus biogenetic beliefs in reducing stigmatization against persons with schizophrenia: an experimental study. J Behav Ther Exp Psychiatry. (2016) 50:231–7. 10.1016/j.jbtep.2015.09.007 26412294

[72] Jorm A Christensen H Griffiths K. The impact of beyondblue: the national depression initiative on the australian public's recognition of depression and beliefs about treatments. Austr N Z J Psychiatry. (2005) 39:248–54. 10.1080/j.1440-1614.2005.01561.x 15777361

[73] Evans-Lacko S Corker E Williams P Henderson C Thornicroft G. Effect of the Time to Change anti-stigma campaign on trends in mental-illness-related public stigma among the English population in 2003-13: an analysis of survey data. Lancet Psychiatry. (2014) 1:121–8. 10.1016/S2215-036670243-3 26360575

[74] Heim E Kohrt B Koschorke M Milenova M Thornicroft G. Reducing mental health-related stigma in primary health care settings in low- and middle-income countries: a systematic review. Epidemiol Psychiatr Sci. (2018) 29:e3. 10.1017/S2045796018000458 30176952 PMC6399081

[75] Clay J Eaton J Gronholm P Semrau M Votruba N. Core components of mental health stigma reductions interventions in low- and middle-income countries: a systematic review. Epidemiol Psychiatr Sci. (2020) 29:e164. 10.1017/S2045796020000797 32883399 PMC7503169

[76] Atal S Foster J. “A women's life is tension”: a gendered analysis of women's distress in poor urban india. Transcult Psychiatry. (2020). 10.1177/1363461520947836. [Epub ahead of print]. 32878592 PMC8060732

[77] McCranie A Recovery in mental illness: the roots, meanings, and implementations of a 'new' services movement. In: Pilgrim D Rogers A Pescosolido B editors. The Sage Handbook of Mental Health and Ilness. London: Sage Publications (2010). p. 471–89. 10.4135/9781446200988.n23

[78] Pescosolido B Medina T Martin J Long J. The “backbone” of stigma: identifying the global core of public prejudice associated with mental illness. Am J Public Health. (2013) 103:853–60. 10.2105/AJPH.2012.301147 23488508 PMC3698809

[79] Markova I Social identities and social representations. In: Moloney G Walker I editors. Social Representations and Identity. New York, NY: Palgrave Macmillan (2007). 10.1057/9780230609181_12

[80] Corrigan P Target-specific stigma change: a strategy for impacting mental illness stigma. Psychiatr Rehabil J. (2004) 28:113–21. 10.2975/28.2004.113.121 15605746

[81] Jackson-Best F Edwards N. Stigma and intersectionality: a systematic review of systematic reviews across HIV/AIDS, mental illness, physical disability. BMC Public Health. (2018) 18:919. 10.1186/s12889-018-5861-3 30049270 PMC6062983

[82] Myers F Woodhouse A Whitehead I McCollam A McBryde L Pinfold V An Evaluation of the Operation of “See Me” the National Campaign against Stigma and Discrimination Associated with Mental Ill Health, Conducted by a Consortium led by the Scottish Development Centre for Mental Health. Edinburgh: Scottish Government Social Research (2009).

[83] Irvine B Billow M Bourgeois M Seeley J. Mental illness training for long term care staff. J Post-Acute Long Term Care Med. (2012) 13:81.e7–13. 10.1016/j.jamda.2011.01.015 21450251 PMC3136641

[84] Hanisch S Twomey C Szeto A Birner U Nowak D Sabariego C. The effectiveness of interventions targeting the stigma of mental illness at the workplace: a systematic review. BMC Psychiatry. (2016) 16:1. 10.1186/s12888-015-0706-4 26739960 PMC4704270

[85] O'Keefe D Message framing variations in health and risk messaging. In: Nussbaum J editor. Oxford Research Encyclopedia. Oxford: Oxford University Press (2017). 10.1093/acrefore/9780190228613.013.308

[86] Jorm A Ross A. Guidelines for the public on how to provide mental health first aid: narrative review. BJPsych Open. (2018) 4:427–40. 10.1192/bjo.2018.58 30450221 PMC6235998

[87] Rossetto A Jorm AF Reavley NJ. Predictors of adults' helping intentions and behaviours towards a person with a mental illness: a six-month follow-up study. Psychiatry Res. (2016) 240:170–6. 10.1016/j.psychres.2016.04.037 27107671

[88] Clement S Lassman F Barley E Evans-Lacko S Williams P Yamaguchi S. Mass media interventions for reducing mental health-related stigma. Cochrane Database Syst Rev. (2013) 7:CD009453. 10.1002/14651858.CD009453.pub2 23881731 PMC9773732

[89] Phelan J. Geneticization of deviant behavior and consequences for stigma: the case of mental illness. J Health Soc Behav. (2005) 46:307–22. 10.1177/002214650504600401 16433278

[90] Read J Haslam N Sayce L Davies E. Prejudice and schizophrenia: a review of the “mental illness is an illness like any other” approach. Acta Psychiatr Scand. (2006) 114:303–18. 10.1111/j.1600-0447.2006.00824.x 17022790

[91] Carter L Read J Pyle M Morrison A. The impact of causal explanations on outcome in people experiencing psychosis: a systematic review. Clin Psychol Psychother. (2017) 24:332–47. 10.1002/cpp.2002 26805779

[92] Hart L Bond K Morgan A Rossetto A Cottrill F Kelly C. Teen mental health first aid for years 7–9: a description of the program and an initial evaluation. Int J Mental Health Syst. (2019) 13:71. 10.1186/s13033-019-0325-4 31788023 PMC6858708

[93] Larkings J Brown P Scholz B “It's often liberating”: consumers discuss causal beliefs in the treatment process. J Mental Health. (2017) 28:397–403. 10.1080/09638237.2017.1417550 29256323

[94] Lam D Salkovskis P. An experimental investigation of the impact of biological and psychological causal explanations on anxious and depressed patients' perception of a person with panic disorder. Behav Res Ther. (2007) 45:405–11. 10.1016/j.brat.2006.03.005 16684503

[95] Clement S Jarrett M Henderson C Thornicroft G. Messages to use in population-level campaigns to reduce mental health-related stigma: consensus development study. Epidemiol Psychiatr Sci. (2010) 19:72–9. 10.1017/S1121189X00001627 20486426

[96] Pescosolido B Phelan J Link B. ‘A disease like any other?’ a decade of change in public reactions to schizophrenia, depression and alcohol dependence. Am J Psychiatry. (2010) 167:1321–30. 10.1176/appi.ajp.2010.09121743 20843872 PMC4429867

[97] Thibodeau R Continuum belief, categorical belief, and depression stigma: correlational evidence and outcomes of an online intervention. Stigma Health. (2019) 5:404–12. 10.1037/sah0000211

[98] Schomerus G Matschinger H Angermeyer M. Continuum beliefs and stigmatizing attitudes towards persons with schizophrenia, depression and alcohol dependence. Psychiatry Res. (2013) 209:665–9. 10.1016/j.psychres.2013.02.006 23465293

[99] Corrigan P Schmidt A Bink A Nieweglowski K Al-Khouja M Qin S. Changing public stigma with continuum beliefs. J Mental Health. (2017) 26:411–8. 10.1080/09638237.2016.1207224 27461413

[100] Meyer J De Ruyter K Grewal D Cleeren K Keeling D Motyka S Categorical versus dimensional thinking: improving anti-stigma campaigns by matching health message frames and implicit worldviews. J Acad Market Sci. (2020) 48:222–45. 10.1007/s11747-019-00673-7

[101] Brewis A Wutich A. Why we should never do it: stigma as a behavior change tool in global health. BMJ Global Health. (2019) 4:1–3. 10.1136/bmjgh-2019-001911 31750003 PMC6830281

[102] Peters G Ruiter R Kok G. Threatening communication: a critical re-analysis and a revised meta-analytic test of fear appeal theory. Health Psychol Rev. (2013) 7:8–31. 10.1080/17437199.2012.703527 23772231 PMC3678850

[103] Orbell S Zahid H Henderson C Changing behavior using the health belief model and protection motivation theory. In: Hagger M Cameron L Hamilton K Hankonen N Lintunen T editors. The Handbook of Behavior Change. Cambridge: Cambridge University Press (2020). p. 46–59. 10.1017/9781108677318.004

[104] Choudhry F Mani V Ming L Kahn T. Beliefs and perception about mental health issues: a meta-synthesis. Neuropsychiatr Dis Treat. (2016) 12:2807–18. 10.2147/NDT.S111543 27826193 PMC5096745

[105] Birtel M Crisp R. “Treating” prejudice: an exposure-therapy approach to reducing negative reactions toward stigmatized groups. Psychol Sci. (2012) 23:1379–86. 10.1177/0956797612443838 23019142

[106] Rusch L Kanter J Angelone A Ridley R The impact of in our own voice on stigma. Am J Psychiatric Rehabil. (2008) 11:373–89. 10.1080/15487760802397660

[107] West K Hewstone M Lolliot S. Intergroup contact and prejudice against people with schizophrenia. J Soc Psychol. (2014) 154:217–32. 10.1080/00224545.2014.888327 24873025

[108] Gillespie A Concluding comment: contact without transformation: The context, process and content of distrust. In: Markova I Gillespie A editors. Trust and Conflict: Representation, Culture and Dialogue. Hove: Routledge (2017). p. 201–16.

[109] Henderson C Stuart H Hansson L. Lessons from the results of three national antistigma programmes. Acta Psychiatr Scand. (2016) 134:3–5. 10.1111/acps.12605 27426640 PMC6680331

[110] Hansson L Stjernsward S Svensson B. Changes in attitudes, intended behavior, and mental health literacy in the swedish population 2009-2014: an evaluation of a national antistigma programme. Acta Psychiatr Scand. (2016) 134:71–9. 10.1111/acps.12609 27426648

[111] Stuart H Chen S.-P. Christie R Dobson K Kirsh B Opening minds in canada: targeting change. Can J Psychiatry. (2014) 59:12–8. 10.1177/070674371405901S05 25565697 PMC4213747

[112] Holland K The unintended consequences of campaigns designed to challenge stigmatizing representations of mental illness in the media. Soc Semiotics. (2012) 22:217–36. 10.1080/10350330.2011.648398

[113] Corrigan P Shaprio J. Measuring the impact of programs that challenge the public stigma of mental illness. Clin Psychol Rev. (2010) 30:907–22. 10.1016/j.cpr.2010.06.004 20674114 PMC2952670

[114] Wong E Collins R Cerully J Roth E Marks J Yu J Effects of stigma and discrimination reduction trainings conducted under the california mental health services authority an evaluation of NAMI's ending the silence. Rand Health Q. (2016) 5:6 10.7249/RR1240 PMC5158208 28083403

[115] Yamaguchi S Wu S.-I. Biswas M Yate M Aoki Y Effects of short-term interventions to reduce mental health-related stigma in university or college students: a systematic review. J Nervous Mental Dis. (2013) 201:490–503. 10.1097/NMD.0b013e31829480df 23719324

[116] Xu Z Rusch N Huang F Kosters M. Challenging mental health related stigma in china: systematic review and meta-analysis. I. interventions among the general public. Psychiatry Res. (2017) 225:449–56. 10.1016/j.psychres.2017.01.008 28780127

[117] Maunder R White F. Intergroup contact and mental health stigma: a comparative effectiveness meta-analysis. Clin Psychol Rev. (2019) 72:101749. 10.1016/j.cpr.2019.101749 31254936

[118] Knaak S Mantler E Szeto A. Mental illness-related stigma in healthcare. Barrier to access and care and evidence-based solutions. Healthc Manage Forum. (2017) 30:111–16. 10.1177/0840470416679413 28929889 PMC5347358

[119] Chen S Koller M Krupa T Stuart H. Contact in the classroom: developing a program model for youth mental health contact-based anti-stigma education. Commun Mental Health J. (2016) 52:281–93. 10.1007/s10597-015-9944-7 26429792

[120] Corrigan P Michaels P Vega E Gause M Larson J Krzyanzowski R. Key ingredients to contact-based stigma change: a cross-validation. Psychiatr Rehabil J. (2014) 37:62–4. 10.1037/prj0000038 24417232

[121] Janoušková M Tušková E Weissová A Trančík P Pasz J Evans-Lacko S. Can video interventions be used to effectively destigmatize mental illness among young people? A systematic review. Eur Psychiatry. (2017) 41:1–9. 10.1016/j.eurpsy.2016.09.008 28049074

[122] Jodelet D Madness and Social Representations: Living with the Mad in One French Community. Hemel Hempstead: Harvester Press (1991)

[123] Arboleda-Florez J Stuart H. Fighting the stigmatization of mental illnesses. Can J Psychiatr. (2012) 57:457–63. 10.1177/070674371205700803 22854027

[124] Harper S Madness, Power and the Media: Class, Gender and Race in Popular Representations of Mental Distress. Basingstoke: Palgrave Macmillan (2009). 10.1057/9780230249509

[125] Maiorano A Lasalvia A Sampogna G Pocai B Ruggeri M Henderson C Contents full article content list abstract background methods results discussion conclusions references accessing resources off campus can be a challenge. lean library can solve it lean library figures & tables article metrics related articles cite. Can J Psychiatry. (2017) 62:702–15. 10.1177/0706743717711172 28622747 PMC5638187

[126] Robinson P Turk D Jilka S Cella M. Measuring attitudes towards mental health using social media: investigating stigma and trivialisation. Soc Psychiatr Psychiatr Epidemiol. (2019) 54:51–8. 10.1007/s00127-018-1571-5 30069754 PMC6336755

[127] Thornicroft A Goulden R Shefer G Rhydderch D Rose D Williams P Newspaper coverage of mental illness in England 2008–2011. Br J Psychiatry. (2013) 202:64–9. 10.1192/bjp.bp.112.112920 23553697

[128] Ross A Morgan A Jorm A Reavley N. A systematic review of the impact of media reports of severe mental illness on stigma and discrimination, and interventions that aim to mitigate any adverse impact. Soc Psychiatr Psychiatric Epidemiol. (2019) 54:11–31. 10.1007/s00127-018-1608-9 30349962

[129] Chamberlain K Lyons A Critical and qualititative approaches to behavior change. In: Hagger M Cameron L Hamilton K Hankonen N Lintunen T editors. The Handbook of Behaviour Change. Cambridge: Cambridge University Press (2020). p. 430–42.

[130] Kelly M Barker M. Why is changing health-related behavior so difficult? Public Health. (2016) 136:109–16. 10.1016/j.puhe.2016.03.030 27184821 PMC4931896

[131] Kalampalikis N Hass V More than a theory: a new map of social thought. J Theory Soc Behav. (2008) 38:449–59. 10.1111/j.1468-5914.2008.00381.x

[132] Foster J. Perpetuating stigma? Differences between advertisements for psychiatric and non-psychiatric medication in two professional journals. J Mental Health. (2010) 19:26–33. 10.3109/09638230902968274 20380495

[133] Morant N. Social representations and professional knowledge: the representation of mental illness among mental health practioners. Br J Soc Psychol. (2010) 45:817–38. 10.1348/014466605X81036 17393882

[134] Rose D Television, madness and community care. J Commun Appl Soc Psychol. (1998) 8:213–28.

[135] Foster J Media presentation of the mental health bill and representations of mental health problems. J Commun Appl Soc Psychol. (2006) 16:285–300. 10.1002/casp.863

[136] Jovchelovitch S. Knowledge in Context. Abingdon, VA: Routledge (2019). 10.4324/9781315173368

[137] Howarth C Foster J Dorrer N. Exploring the potential of the theory of social representations in community-based health research—and vice versa? J Health Psychol. (2004) 9:229–43. 10.1177/1359105304040889 15018725

[138] Bhavsar V Schofeild P Das-Munshi J Henderson C. Regional differences in mental health stigma—Analysis of nationally representative data from the health survey for England, 2014. PLoS ONE. (2019) 14:e0210834. 10.1371/journal.pone.0210834 30668597 PMC6342445

[139] Rose D Efraim D Gervais M Joffe H Jovchelovitch S Morant N Questioning consensus in social representations theory. Papers Soc Rep. (1995) 4:150–76.

[140] Foster J Unification and differentiation: a study of the social representations of mental illness. Papers Soc Rep. (2001) 10:31–18.

[141] Staerkle C Clemence A Spini D Social representations: a normative and dynamic intergroup approach. Polit Psychol. (2011) 32:759–68. 10.1111/j.1467-9221.2011.00839.x

[142] Moscovici S The Invention of Society: Psychological Explanations for Social Phenomena. Trans. by Halls WD. Polity Press (1993).

[143] Joffe H Thematic analysis. In: Harper D Thompson A editors. Qualititative Research Methods in Mental Health and Psychotherapy: A Guide for Students and Practioners. Chichester: Wiley-Blackwell (2012). p. 209–23. 10.1002/9781119973249.ch15

[144] Foster J Performance in bethlem, fulbourn and brookwood hospitals: a social psychological and social historical examination. In: Harpin A Foster J editors. Performance, Madness and Psychiatry. Abingdon, VA: Routledge (2014). p. 42–6. 10.1057/9781137337252_3

[145] Moon G Kearns R Joseph A The Afterlives of the Psychiatric Asylum: the Recycling of Concepts, Sites and Memories. Farnham: Ashgate (2015). 10.4324/9781315612317

[146] Reali F Soriano T Rodriguez D How we think about depression: the role of linguistic framing. Revista Latinoamericana de Psicol. (2015) 48:127–36. 10.1016/j.rlp.2015.09.004

[147] Yanni C The Architecture of Madness. Minneapolis, MN: University of Minnesota Press (2007)

[148] Arthi Representing mental illness: a case of cognitive polyphasias. Pap Soc Rep. (2012) 21:5.1–5.16.

[149] Wagner W Duveen G Verma J Themel M 'I have some faith and at the same time I don't believe' - cognitive polyphasia and cultural change in India. J Commun Appl Soc Psychol. (2000) 10:301–14. 10.1002/1099-1298(200007/08)10:4<301::AID-CASP585>3.0.CO;2-V

[150] Provencher C Towards a better understanding of cognitive polyphasia. J Theor Soc Behav. (2011) 41:377–95. 10.1111/j.1468-5914.2011.00468.x

[151] Marchetti I Koster E. Brain and intersubjectivity: a Hegelian hypothesis on the self-other neurodynamics. Front Hum Neurosci. (2014) 8:11. 10.3389/fnhum.2014.00011 24478677 PMC3901003

[152] Markova I The epistemological significance of the theory of social representations. J Theor Soc Behav. (2008) 381:461–87. 10.1111/j.1468-5914.2008.00382.x

[153] Moreno-Kustner B Martin C Pastor L. Prevalence of psychotic disorders and its association with methodological issues. a systematic review and meta-analyses. PLoS ONE. (2018) 13:e0195687. 10.1371/journal.pone.0195687 29649252 PMC5896987

[154] Van Os J Linscott R Myin-Germeys I Delespaul P Krabbendam L A systematic review and meta-analysis of the psychosis continuum: evidence for a psychosis proneness-persistence-impairment model of psychotic disorder. Psychol Med. (2009) 39:179–95. 10.1017/S0033291708003814 18606047

[155] Smith N O'Connor C Joffe H Social representations of threatening phenomena: the self-other thema and identity protection. Papers Soc Rep. (2015) 24:1–23.

[156] Sontag S. Illness as Metaphor. New York, NY: First Printing (1978).

[157] Rossetto A Jorm A Reavley N. Developing a model of help giving towards people with a mental health problem: a qualitative study of mental health first aid participants. Int J Mental Health Syst. (2018) 12:48. 10.1186/s13033-018-0228-9 30151033 PMC6102795

[158] Crawford R. The boundaries of the self and the unhealthy other: reflections on health, culture and AIDS. Soc Sci Med. (1994) 38:1347–65. 10.1016/0277-9536(94)90273-9 7517576

[159] Weiner B Perry R Magnusson J. An attributional analysis of reactions to stigmas. J Personal Soc Psychol. (1988) 55:738–48. 10.1037/0022-3514.55.5.738 2974883

[160] Schnittker J. An uncertain revolution: why the rise of a genetic model of mental illness has not increased tolerance. Soc Sci Med. (2008) 67:1370–81. 10.1016/j.socscimed.2008.07.007 18703264

[161] Scull A Madness in Civilization. London: Thames & Hudson Ltd (2020).

[162] Lupton D Sociology risk. In: Mythen G Walklate S editors Beyond the Risk Society: Critical Reflections on Risk and Human Security. Maidenhead: Open University Press (2006). p. 11–24.

[163] Idoiaga N de Montes LG Asla N Larrañaga M Where does risk lie in sexual practices? A study of young people's social representations. Health Risk Society. (2020) 22:3–4; 249–265. 10.1080/13698575.2020.1793304

[164] Kearney J Donovan C (editors). Introduction: identities, individuals and theories of risk. In: Constructing Risky Identities in Policy and Practice. London: Palgrave Macmillan 10.1057/9781137276087_1

[165] Goodwin R Kwiatkowska A Realo A Kozlova A Nguyen Luu LA Nizharadze G. Social representations of HIV/AIDS in five Central European and Eastern European countries: a multidimensional analysis. AIDS Care. (2004) 16:669–80. 10.1080/09540120412331269521 15370056

[166] Yang L Pearson V Understanding families in their own context: Schizophrenia and structural family therapy in Beijing. J Fam Ther. (2002) 24:233–57. 10.1111/1467-6427.00214

[167] Potts L Henderson C. Moderation by socioeconomic status of the relationship between familiarity with mental illness and stigma outcomes. SSM Popul Health. (2020) 11:1–8. 10.1016/j.ssmph.2020.100611 32715077 PMC7378684

[168] Fernandez M Ruiter R Markham C Kok G. Intervention mapping: theory- and evidence-based health promotion program planning: perspective and examples. Front Public Health. (2019) 7:209. 10.3389/fpubh.2019.00209 31475126 PMC6702459

[169] Ogden J Celebrating variability and a call to limit systemization: the example of the behavior change technique taxonomy and the behavior change wheel. Health Psychol Rev. (2016) 10:245–50. 10.1080/17437199.2016.1190291 27189585

[170] Springer A Evans A Ortuno J Salvo D Arevalo M. Health by design: interweaving health promotion into environments and settings. Fronti Public Health. (2017) 5:268. 10.3389/fpubh.2017.00268 29043248 PMC5632521

[171] Hudson J Moon Z Hughes L Moss-Morris R Engagement of stakeholders in the design, evaluation, implementation of complex interventions. In: Hagger M. Cameron L Hamilton K Hankonen N Lintunen T editors. The Handbook of Behaviour Change. Cambridge: Cambridge University Press (2020). p. 349–60. 10.1017/9781108677318.024

[172] O'Connor C Embodiment and the construction of social knowledge: towards an integration of embodiment and social representations theory. J Theor Soc Behav. (2016) 47:2–24. 10.1111/jtsb.12110

[173] Kok G Peters L Ruiter R. Planning theory- and evidence-based behavior change interventions: a conceptual review of the intervention mapping protocol. Psicol Reflex Crít. (2017) 30:19. 10.1186/s41155-017-0072-x 32026109 PMC6975763

[174] Peters L Kok G Ten Dam G Buijs G Paulussen T. Effective elements of school health promotion across behavioral domains: a systematic review of reviews. BMC Public Health. (2009) 9:182. 10.1186/1471-2458-9-182 19523195 PMC2702385

[175] Kemp C Jarrett B Kwon C Song L Jette N Sapag J. Implementation science and stigma reduction interventions in low- and middle-income countries: a systematic review. BMC Med. (2019) 17:6. 10.1186/s12916-018-1237-x 30764820 PMC6376798

[176] Flick U Foster J Caillaud S Researching social representations. In: Sammut G Andreouli E Gaskell G Valsiner J editor. The Cambridge Handbook of Social Representations. Cambridge: Cambridge University Press (2015). p. 64–82. 10.1017/CBO9781107323650.007

[177] Cresswell T Geographic Thought: A Critical Introduction. Chichester: John Wiley & Sons (2013)

[178] Foster J. Beyond otherness: controllability and location in mental health service clients' representations of mental health problems. J Health Psychol. (2003) 8:632–44. 10.1177/13591053030085012 19177722

[179] Joffe H Perez-Fuentes G Potts H Rossetto T How to increase earthquake and home fire preparedness: the fix-it intervention. Nat Hazards. (2016) 84:1943–65. 10.1007/s11069-016-2528-1

[180] Joffe H Rossetto T Solberg C O'Connor C Social representations of earthquakes: a study of people living in three highly seismic areas. Earthquake Spectra. (2013) 29:367–97. 10.1193/1.4000138

[181] Joffe H Potts H Rossetto T Dogulu C Gul E Perez-Fuentes G. The Fix-it face-to-face intervention increases multihazard household preparedness cross-culturally. Nat Hum Behav. (2019) 3:453–61. 10.1038/s41562-019-0563-0 30936428

[182] GASA GASA: Global Anti-Stigma Alliance. London: Time to Change (2017).

[183] Stuart H. Reducing the stigma of mental illness. Glob Mental Health. (2016) 3:e17. 10.1017/gmh.2016.11 28596886 PMC5314742

